# Clinical Presentation and Microarray Analysis of Peruvian Children with Atypical Development and/or Aberrant Behavior

**DOI:** 10.1155/2014/408516

**Published:** 2014-10-20

**Authors:** Merlin G. Butler, Kelly Usrey, Jennifer L. Roberts, Stephen R. Schroeder, Ann M. Manzardo

**Affiliations:** ^1^Department of Psychiatry and Behavioral Sciences, The University of Kansas Medical Center, 3901 Rainbow Boulevard, MS 4015, Kansas City, KS 66160, USA; ^2^Kansas University Center on Development Disabilities, Schiefelbusch Institute for Life Span Institute, The University of Kansas, Lawrence, KS 66045, USA; ^3^Department of Pediatrics, The University of Kansas Medical Center, Kansas City, KS 66160, USA

## Abstract

We report our experience with high resolution microarray analysis in infants and young children with developmental disability and/or aberrant behavior enrolled at the Centro Ann Sullivan del Peru in Lima, Peru, a low income country. Buccal cells were collected with cotton swabs from 233 participants for later DNA isolation and identification of copy number variation (deletions/duplications) and regions of homozygosity (ROH) for estimating consanguinity status in 15 infants and young children (12 males, 3 females; mean age ± SD = 28.1 m ±  7.9 m; age range 14 m–41 m) randomly selected for microarray analysis. An adequate DNA yield was found in about one-half of the enrolled participants. Ten participants showed deletions or duplications containing candidate genes reported to impact behavior or cognitive development. Five children had ROHs which could have harbored recessive gene alleles contributing to their clinical presentation. The coefficient of inbreeding was calculated and three participants showed first-second cousin relationships, indicating consanguinity. Our preliminary study showed that DNA isolated from buccal cells using cotton swabs was suboptimal, but yet in a subset of participants the yield was adequate for high resolution microarray analysis and several genes were found that impact development and behavior and ROHs identified to determine consanguinity status.

## 1. Introduction

Research involving early recognition of developmental/intellectual disabilities with or without aberrant behaviors such as self-injury, aggression, stereotypic patterns, and autism is on the increase but the causation is poorly understood. Advances in genetic testing now allow for the identification of genetic disturbances including chromosomal abnormalities and gene mutations or variants [1]. Most recently, the rise of chromosomal microarrays or comparative genomic hybridization arrays (aCGH) is increasingly helpful for determination of copy number variants (CNV) by identifying subtle DNA deletions and duplications that contribute significantly to genomic variation (e.g., 21% of consecutive patients with autism or intellectual disability presenting for genetic services at an academic setting) [2].

Single nucleotide polymorphisms (SNPs) are also helpful in detecting regions of homozygosity [large genomic regions without SNPs which may indicate gene alleles identical by descent from a common ancestor with evidence of consanguinity or due to areas of loss of heterozygosity (e.g., uniparental maternal disomy of chromosome 15 in which both chromosome 15s are inherited from the mother causing Prader-Willi syndrome, a classical genetic disorder due to errors in genomic imprinting)] [3–5]. Consanguinity is a common occurrence in some cultures and offspring of consanguineous or related couples sharing gene alleles are more likely to inherit mutant alleles for disease, particularly autosomal recessive conditions. The working definition of consanguinity is a union between individuals whom are biologically related as second cousins or closer [6]. Offspring of consanguineous parents also have increased risks for birth defects and multifactorial disorders [7]. The level of consanguinity in an individual can be empirically measured and quantified as the coefficient of inbreeding (*F*) based upon the level of homozygosity in the genome and assessed using SNP data from microarray analysis [3]. Couples related as second cousins or closer (*F* ≥ 0.0156) and their progeny account for an estimated 10% of the global population [6]. In Peru, the rate of consanguineous marriages is estimated to be between 1% and 4% [6].

Numerous studies have applied molecular karyotyping with microarray analysis in identifying subtle DNA changes to explain the causation of unexplained developmental/intellectual disabilities, autism spectrum disorders, and multiple congenital anomalies. Microarrays can identify submicroscopic deletions and duplications not found by routine G-banded chromosome studies. Chromosomal microarray analysis is now considered in the first tier of clinical genetic testing in those patients presenting with the above common reasons for referral [8]. The testing is readily available in most genetic centers in high income or industrialized societies, but yet many patients in underprivileged or low income countries do not have readily available access to these genetic services. Hence, the utility of microarray studies in infants and children characterized with atypical development has not been established in low income or poor countries.

Herein, we report our genetics experience with high resolution microarrays in individuals after 6 months of age from Peru, a low income country where these services are limited, and those at risk for developmental disability, autism, or aberrant behavior. The goals of our study consisted of the following: (1) to identify, screen, enroll, and assess Peruvian infants and children at a young age with atypical development or with behavioral concerns for high resolution microarray analysis to study copy number variation and consanguinity status and to generate clinical case reports and (2) to test whether an adequate DNA yield can be obtained from buccal cells collected with cotton swabs only and stored at room temperature until shipped to the USA for DNA isolation.

## 2. Subjects and Methods

Participants were recruited as part of an innovative national outreach effort to identify and treat children with developmental problems in urban, rural, underprivileged, or otherwise underserved regions of Peru. Participants were solicited by approved television, radio, and newspaper announcements throughout Peru, requesting parents to respond if they had a child between the ages of 6 to 36 months and concerns regarding their child's development, cognition, communication, behavioral problems especially self-injury (SIB), stereotypy and aggression, or a family history of disabilities, medical conditions, or issues related to environmental or other unexplained factors. The parents who responded to the announcements called the Telephone Triage Service at Centro Ann Sullivan del Peru (CASP), a developmental training center affiliated with the University of Kansas and located in Lima. After brief discussion of their appropriateness for the project, 341 respondents were invited to CASP for a screening interview by trained interviewers/examiners, using the Parental Concerns Questionnaire (PCQ), a checklist based upon risk factors related to behavioral problems among people with developmental disabilities (DD) [9]. If parents responded “yes” to any of these risk factors, they were included in the study. After screening, 262 families were invited to attend the CASP and 233 agreed to undergo cognitive and communication assessments, developmental pediatric and dental exams and visual, auditory, and behavioral evaluations performed by 10 teams of personnel trained and monitored by eight consultants from the USA who were experts in their respective disciplines (summarized by Mayo-Ortega et al.) [10]. The parents of all participants signed consent forms approved by the Human Subjects Institutional Review Board Committees at CASP and the Human Subjects Committee at the University of Kansas prior to evaluation.

Participants were screened using the Parent Concerns Questionnaire, a 15-item yes/no checklist based on an extensive list of 53 developmental risk factors for severe behavioral problems in children across nine domains [10]. The domains of concern were intellectual disability; communication impairment; genetic syndromes; family history of brain disorders; several medical conditions; psychiatric factors; neurochemical and metabolic factors; neuropsychological factors; and developmental motor factors.

Each participant received (1) anthropometric measures of height, weight, and head circumference; (2) developmental pediatric exam with consults by local Peruvian specialists from pediatric neurology, medical genetics, nutrition, and child psychiatry as needed. If the pediatricians suspected autism, the Child Autism Rating Scale (CARS) was completed by the parents [11]; (3) behavior was assessed using the Behavior Problems Inventory (BPI-01) as the primary instrument [12]; (4) cognitive assessment was undertaken using the Bayley Scales of Infant Development, Third Edition (BSID III) [13]; (5) communication was assessed using the Communication and Symbolic Behavior Scales (CSBS) [14]; (6) visual assessment was performed by a pediatric ophthalmologist from the USA; (7) hearing assessment was performed by a hearing specialist from the USA; and (8) dental assessment was performed by a Peruvian odontologist. The latter three assessments were performed by experts in treating people with DD.

Both the BSID and CSBS are standardized measures with a standard deviation of 15. Both scales are designed for the age group under study. One standard deviation below the mean of 100 for these instruments is a commonly accepted cut-off considered to be at-risk for developmental delay. Two standard deviations are often defined as intellectual or communication disability along with a measure of social performance ability. A standard cut-off score for autism on the CARS is 35 but used as a screening measure only.

Four cotton swabs that were readily available locally were used to collect buccal cells from each child by the medical examiner as part of a physical examination. Saliva collection kits for DNA isolation were not available locally. Parents were not available for specimen collection. Swabs were then stored at room temperature in a dry location for DNA isolation and genetic testing to be performed later in the USA according to protocols reported by Rethmeyer et al. [15]. For this preliminary study, DNA was isolated from 111 samples which represented about one-half of the 233 participants from which the buccal samples were collected. These DNA samples were then monitored for sufficient quantity (e.g., 3 *μ*g) and quality [i.e., intact, nondegraded single DNA band by gel electrophoresis with an acceptable optic density spectroscopic reading (e.g., 1.7)] required for high resolution microarray analysis. Approximately 50% of the DNA samples obtained did not meet the laboratory criteria required for high resolution microarray hybridization, reflecting the buccal swab collection process, handling, and storage prior to DNA isolation. Participants were excluded from microarray analysis with an inadequate DNA yield or with a known cause of their atypical development and/or aberrant behavior (e.g., Down syndrome, neurofibromatosis, fragile X syndrome, pre- or postnatal trauma, infections, and hydrocephalus or brain malformations) after review of medical, laboratory, and brain imaging records (if available) and clinical assessments with examinations performed by local medical experts and specialists. Of the approximately 50 DNA samples meeting laboratory criteria for microarray analysis, funding was available to analyze 15 representative children (12 males: 3 females, mean age ± SD = 28.1 m ± 7.9 m; age range 14 m–41 m; see Table 1) selected at random from those not having a known cause for their atypical development and/or aberrant behavior.

## 3. Results

### 3.1. High Resolution Microarray Hybridization and Analysis

Two high resolution microarray platforms widely utilized in the clinical setting for patients in higher income countries were chosen for our study participants from Peru. Five DNA samples were analyzed at the University of Kansas Medical Center Genomics Core Facility using the Affymetrix Genome-wide Human SNP Array 6.0 version utilizing 1.8 million copy number variant (CNV) and single nucleotide polymorphism (SNP) DNA probes. The remaining 10 samples were analyzed by iLife Discoveries (Haryana, India) using the Affymetrix Cytoscan HD Array utilizing 2.3 million copy number variant (CNV) and single nucleotide polymorphism (SNP) DNA probes (Affymetrix, Santa Clara, CA). The Affymetrix Chromosome Analysis Suite 1.2.2 software version was used for determination of the number, location, and size of the CNV (i.e., deletions or duplications) or regions of homozygosity (ROH) by downloading and analyzing the microarray genome-wide SNP and CNV probe data on each participant and following standards recommended by the manufacturer of the high resolution microarrays for determination of genetic lesions and copy number variation. Deletions (losses) and duplications (gains) in the genome were based on the number of DNA probes deleted (or duplicated) over and within an established genomic distance and the number of involved DNA markers. We chose 100 kb size gains and losses across the genome with a minimum of 50 markers to make a call for a genomic abnormality. For regions of known significance in the human genome, a minimal setting of 25 kb in size and 50 markers for gains and losses were needed. For ROH, a minimal setting of 3 to 5 Mb was utilized.

In lieu of family history or pedigree analysis, we also estimated consanguinity and the coefficient of inbreeding (*F*) was calculated based on ROHs for each participant using the model proposed by Kearney et al. [3]. The coefficient of inbreeding considers regions of homozygosity from autosomal chromosomes greater than 3 Mb in size. Sex chromosomes are excluded due to limited recombination of the X chromosome. The sum of ROHs greater than 3 Mb from autosomal chromosomes is divided by the total length of autosomal chromosomes in humans (equal to 2,867,733 kb or 2,867.733 Mb in size; using the Human Genome Browser—hg 18 assembly—http://www.genome.ucsc.edu/index.html) [16] to estimate the percentage of inheritance by descent or consanguinity relationship. The predicted percentage of genetic material identical by descent for an individual can then be assessed for various degrees of relationship (e.g., 25% for first degree relatives (parents, children, and siblings) in which one-fourth of the genes are identical by descent with regions of homozygosity determined by lack of DNA polymorphisms (*F* = 0.25); 12.5% for second degree relatives (grandparents, grandchildren, half-siblings, uncles, aunts, nieces, and nephews) (*F* = 0.125); 6% for third-degree relatives (first cousins) (*F* = 0.0625)). Couples related as fifth degree relatives (second cousins or closer) with an *F* ≥ 0.0156 are considered consanguineous [6].

### 3.2. Clinical Presentations

Clinical presentation of the 15 children studied included development delay or aberrant behavior as characterized by interdisciplinary assessments listed in Table 1. Only one child (participant 21) had a normative score for both intelligence and communication ability. Three children (participants 144, 146, and 188) were untestable because of the severity of their intellectual disability. Six children had high CARS scores while the remaining children were not suspected of having autism by the developmental pediatricians.

Assessments for SIB, stereotyped behavior, and aggression were performed with the BPI-01 instrument. Normative data showed that 10 of the 15 children (participants 21, 32, 44, 94, 102, 120, 146, 176, 218, and 237) had high scores according to Rojahn et al. [17]. These observations were especially true for both SIB and aggression and much less for stereotyped behavior. The interdisciplinary assessments and their relation to risk factors for behavioral problems used in our study are described elsewhere [9].

The microarray genetic findings of the 15 children with atypical development and autism or aberrant behavior are summarized in Table 2. Participant 21 was a male presenting at 28 months of age with atypical development. He was large for his age and had normal cognition but with increased stereotypical behavior and autistic features. He had a 7q22.3 deletion (231 kb in size). This small deletion included the* COG5 *gene which is located in the autism susceptibility 1 (AUTS1) locus known to cause atypical development and autism [18] supporting an underlying cause of his developmental/behavioral problems.

Participants 32 and 44 are both males at 41 months and 36 months of age, respectively, and presented with normal cognition. The CARS and BPI scores supported the diagnosis of autism. Participant 32 had a 5p14.1 deletion including the* CDH9 *gene, one of the cadherin gene members reported to play a role in autism and supported by genome-wide association studies indicating this genomic region to be highly associated with autism [19]. Participant 44 showed disturbed* NDUFS7 *and* GAMT* genes due to a chromosome 19p13.3 duplication (1043 kb in size). These genes may cause developmental delay, psychomotor retardation, hypotonia, regression, and metabolic problems [20, 21]. He also had a 217 kb deletion of chromosome 1p12-p11.2 including the* NOTCH2* gene. Notch receptor defects can cause failure to thrive, renal abnormalities, heart defects, and learning disabilities [22] and may have contributed to his clinical findings. The coefficient of inbreeding for participant 44 indicated that the parents were unrelated.

Participant 94 is a 19-month-old male with several microarray findings including a very small 9q22.31 partial duplication (52 kb in size) including the* ROR2* gene which encodes a receptor tyrosine kinase when defective causes Robinow syndrome. This syndrome is characterized by short stature, mesomelia, brachydactyly, a particular facial appearance, and developmental delay [23]. He showed high BPI and CARS scores representing behavioral problems and autism but had normal stature and no dysmorphic features. In addition, a very small partial 16p13.3 duplication was found including the* CACNA1H* gene encoding a calcium ion channel subunit, causing susceptibility to epilepsy [24]. A partial duplication of the* WDR45* gene was also found at Xp11.23, which regulates the assembly of multiprotein complexes and protein-protein interactions. Truncating and missense mutations of this gene have been implicated in neurodegeneration with brain iron accumulation leading to intellectual disability, dementia, dystonia, and tremors [25]. In addition, the* SYP* gene was also duplicated at Xp11.23 and is an integral membrane protein that regulates synaptic vesicle endocytosis associated with X-linked intellectual disability and epilepsy [26]. The coefficient of inbreeding indicates that the parents were unrelated.

Participant 102 is a 28-month-old male with tall stature and microcephaly with a very small gain of the 22q11.21 region containing the* PRODH* gene encoding proline dehydrogenase. This enzyme catalyzes the conversion of proline to pyrroline-5-carboxylate with disturbances linked to the susceptibility of schizophrenia, autism, hyperactivity, aggression, and intellectual disability [27]. He had BSID, BPI, CSBS, and CARS scores indicative of developmental delay, behavioral and communication problems, and autism. The coefficient of inbreeding indicated that the parents were unrelated.

Participant 113 is a 26-month-old female with an ROH at Xq21.33-q22.1 including the* PCDH19* and* SRPX2 *genes.* PCDH19* encodes protocadherin 19 known to play a role in autism while the* SRPX2* gene is associated with Rolandic epilepsy, intellectual disability, and speech dyspraxia [28]. She had CSBS and CARS scores indicating communication problems and autism. Additional ROHs at Xq11.1-q12 and Xp11.22-p11.21 included genes (e.g.,* OPHN1*) when defective are implicated in the development of intellectual disabilities. The* OPHN1 *geneencodes the Rho GTPase-activating protein associated with X-linked intellectual disabilities and cerebellar hypoplasia [29]. The coefficient of inbreeding represented a second-third cousin relationship.

Participant 120 is a 36-month-old male who showed a very small gain at 22q11.21 which contains the* RTN4R* gene for susceptibility to schizophrenia by encoding a protein component of myelin and preventing axonal regeneration [30]. His BPI scores indicated a high level of aggression. The coefficient of inbreeding represented a first-second cousin relationship, indicating consanguinity.

Participant 146 is a 34-month-old male with growth retardation and showed an ROH identified at 7q31.1-q31.2 which contains the* FOXP2* gene encoding forkhead box P2, a putative transcription factor and DNA binding domain. Mutations leading to haploinsufficiency of this gene can result in speech-language disorder-1 [31]. His CSBS score indicated impaired communication and symbolic behavior skills. The coefficient of inbreeding indicated that the parents were unrelated.

Participant 165 is a 32-month-old female with a 20q13.2-q13.3 deletion (7,339 kb in size) involving* GNAS, MC3R, CDH6, TFAP2C, *and approximately 50 other genes.* GNAS *beinga complex locus containing four genes with three of the genes (*XLAS, NESP, and NESPAS*) being imprinted or expressed differently depending on the parent of origin (reviewed by Butler [32]). The* MC3R* (melanocortin 3 receptor) gene is known to play a role in obesity while the* CDH4* is a member of the cadherin gene family mediating calcium-dependent cell-cell adhesion and plays a role in autism.* TFAP2C* modulates the transcriptional activity of vitamin A and target genes via the retinoic receptors and is expressed in migrating neural crest cells in the developing embryo [33]. She presented with multiple congenital anomalies, generalized growth retardation, developmental delay, and impaired communication and symbolic behavior skills. The coefficient of inbreeding represented a first-second cousin relationship, indicating consanguinity.

Participant 176 is a 37-month-old male with ROHs noted on chromosomes 3, 17, and 22 within regions where several genes (i.e.,* POU1F1, RAI1, and EP300*) associated with intellectual disability are found. If mutated and inherited in a recessive pattern then intellectual disability, hypotonia, speech delay, self-injury, microcephaly and behavioral problems can occur [34–36]. Several of these features were seen in this participant including Moebius syndrome, microcephaly, developmental delay, stereotypy, and impaired communication and symbolic behavior skills. The coefficient of inbreeding represented a second-third cousin relationship.

Participant 178 is a 14-month-old male with macrocephaly and two ROHs on chromosomes 2 and 11 involving the* HSPD1, PACS1, NDUFV1, and NDUFS8* genes that may play a role in psychomotor delay, hypotonia, and atypical development [37–40]. He did present with atypical development. The coefficient of inbreeding represented a second-third cousin relationship.

Participant 188 is a 28-month-old female who was small for age with microcephaly and showed a 1q21.1 duplication (1,826 kb in size) which included 25 genes. Duplications of this chromosome region have been reported previously in individuals with mild to moderate global delay, abnormal head size, autism, self-injury, stereotypy, speech delay, and mild facial dysmorphism [41]. In addition, this participant also had a very small 22q11.21 duplication including the* PRODH* and* RTN4R *genes, as stated previously both reportedly play a role in schizophrenia [27, 30]. She presented with atypical development and impaired communication and symbolic behavior skills. The coefficient of inbreeding represented a first-second cousin relationship, indicating consanguinity.

Participant 218 is a 23-month-old male who showed a very small gain at 3q26.31 representing a partial duplication of the* NLGN1* gene which encodes a neuroligin. Neuroligins function as ligands for neurexins or cell-surface receptors. The neurexin/neuroligin complex is Ca(2+)-dependent and present at synapses in the central nervous system and required for efficient neurotransmission involved in the formation of synaptic contacts proposed to cause autism [42, 43]. In addition, ROHs at 9q21.33-q22.2 and 17q22-q23.2 were found and contained genes (*NTRK2, MKS1*) that may play a role in obesity and developmental or speech delay [45, 46]. He presented with atypical development. The coefficient of inbreeding indicates that the parents were unrelated.

Participant 237 is a 21-month-old male who was large for age and showed a gain at 7p22.3 which includes the* FAM20C* gene associated with Raine syndrome. This syndrome is characterized by short stature, microcephaly, craniofacial dysplasia, renal abnormalities, developmental delay, and self-stimulating behavior [47]. In addition, gains of genetic information on the X chromosome seen in the participant included several genes involved with muscle development and learning problems. These disturbances could explain the atypical development and behavior problems seen at an early age in our participant although other features of Raine syndrome were not present.

## 4. Discussion

Chromosomal microarray analysis is now considered in the first tier of clinical genetic testing when evaluating children presenting with unexplained developmental delay/intellectual disabilities, autism spectrum disorders, and/or multiple congenital anomalies. This method yields a much higher result than routine chromosome studies by detecting small submicroscopic deletions and duplications in the genome. Our study is the first to undertake advanced genetic testing using high resolution microarray technology at such a young age (i.e., in infants) systematically ascertained and assessed for atypical development and aberrant behavior from an underserved population in a low income country (Peru). We utilized locally available cotton swabs as salvia collection kits with DNA preservative were not accessible to collect buccal cells for later use in microarray hybridization. DNA collected from approximately one-half of the buccal cell samples was acceptable for microarray analysis indicating an overall suboptimal yield. Several previous studies using chromosomal microarrays have shown a diagnostic yield of 11% to 20% with clinically significant CNVs in those individuals presenting with unexplained intellectual disabilities in high income countries (e.g., in the USA) [2], while a yield of 16% was found in a cohort of Greek children with developmental delay [44].

Detection of regions of homozygosity (ROH) with microarray technology in itself is not diagnostic for an underlying condition and may have no clinical outcome. However, when present in multiple areas throughout the genome versus long stretches of homozygosity confined to a single chromosome, then these regions may be due to identical by descent or from a common (or shared) ancestor raising the possibility that mutant recessive gene alleles if present in these regions could lead to genetic disorders. If these regions are large (e.g., >3 Mb), then the likelihood increases for inheriting a genetic disorder with clinical consequences [3]. ROHs less than 10 Mb are relatively common and likely result from a common ancestor but should not be overlooked when examining for genes in these regions causing atypical development and/or aberrant behavior impacting neurological development or function.

When parents of a child share a recent common ancestor then this relationship becomes consanguineous in nature with the closer relationship having a greater likelihood in producing a child with two copies of a deleterious recessive gene allele mutation received from the parents. Conversely, if one or more regions show extended homozygosity particularly longer than 10 Mb in size on a single chromosome, then the most likely cause is uniparental disomy with loss of heterozygosity of the entire chromosome (error in meiosis II) or as the segmental type (reviewed by Papenhausen et al. [4]). This event is supported by SNP probe data without evidence of polymorphisms within the involved regions and with CNV data showing a normal or nondeletion status for the specific chromosome region or conversely the entire chromosome.

Unfortunately, no data were available from the parents. The detection of high levels of ROH without parental data limits accurate interpretation for consanguinity and one can only conclude that consanguinity is present in the community in which the participant lives. Any follow-up study should include parents and demographic survey with questions on family structure/consanguinity and socioeconomic status.

Of the 15 children selected at random to study with microarray analysis, 10 had genomic duplications or deletions containing genes when defective are known to be associated with neurodevelopment and function including autism, behavior problems, schizophrenia, and developmental delay. Therefore, these rearrangements harboring genes become candidates for contributing to the clinical presentations observed in our participants. Two participants had identifiable microduplication/deletion syndromes including participant 165 with a 20q13.2-q13.33 deletion encompassing the complex GNAS locus and other genes within the region (see Table 2). This deletion has previously been reported in conjunction with intellectual disability, severe pre- and postnatal growth retardation, facial dysmorphism, mild psychomotor retardation, and hypotonia [33]. Participant 188 was identified as having a 1q21.1 duplication, repeatedly identified in patients with autism and/or intellectual disability. Hence, four of the 15 children had a deletion involving four different chromosomes with deletions ranging from a small 217 kb to a large 7,338 kb size. Seven of the 15 children were identified with 11 duplications involving eight different chromosomes and the duplications ranged from a very small 52 kb to a large 7,247 kb size. The deletions containing genes identified in participants 21, 32, 44, and 165 likely accounted for their clinical presentations and similarly with chromosomal duplications found in seven of the children (participants 44, 94, 102, 120, 188, 218, and 237). Very small duplications or deletions in the genome particularly those <200 kb in size should be considered preliminary without further characterization of the genes involved in the respective disturbed genomic regions. Whether the reported findings would impact the phenotype or likely be pathogenic would require more testing which is beyond the scope of this study.

Five of the children (participants 113, 146, 176, 178, and 218) in our study had ROHs which may have contributed to their clinical presentation if disturbed recessive candidate gene alleles were present in the extended stretches of homozygosity and thus these participants would have received two copies. The ROHs ranged from 3,018 to 6,237 kb in size. Three children did not have sufficient SNP data for calculation of the coefficient of inbreeding to determine inbreeding status (no ROHs seen). Six participants were found to be unrelated while three showed ROHs consistent with a first-second cousin relationship (consanguineous) but had recognized chromosomal structural anomalies as well including genes associated with developmental and/or behavioral problems. Three participants showed a second-third cousin relationship but limitations in our study prevented detailed family history and parental physical examinations with collection of specimens for parental laboratory evaluations in order to analyze CNVs or SNPs for determining familial versus* de novo* CNV status or consanguinity. Limitations in our study included lack of resources and access of parental data and laboratory evaluations to analyze CNVs.

In summary, our study showed that in less developed countries high resolution microarray analysis can be done where blood collection and specimen processing may be limited using locally available cotton swabs for buccal cell collection without a DNA preservative and storage at room temperature. However, as expected, the capacity to provide adequate quantity and quality of DNA was suboptimal with about one-half of the specimens yielding DNA suitable for high resolution microarray analysis. Saliva is now becoming a more common specimen source for collection of DNA for genetic testing particularly for the pediatric age group and the use of a DNA preservative. Two-thirds of our participants showed DNA copy number variation consistent with deletions or duplications in genomic regions containing known or candidate genes for atypical development or behavioral problems. One-fifth of the study participants showed evidence of consanguinity but parental samples were not available. We conclude that advanced genetic testing can yield meaningful data even in suboptimal sample collection conditions with limited resources.

## Figures and Tables

**Table 1:** Clinical characteristics and assessment scores of selected subjects for microarray analysis.

Subject ID	Reason for referral	Gender	Age (mo)	Ht cm (%ile)	Wt kg (%ile)	HC cm (%ile)	BSID	BPI Total	BPI SIB	BPI Stereo.	BPI Aggr.	CSBS	CARS
21	Atypical development	M	28	98 (99)	16.1 (96)	53 (98)	100	54	11	24	19	115	34
32	Autism	M	41	98 (50)	16.0 (70)	50 (50)	80	28	7	21	0	76	37
44	Autism	M	36	96 (60)	14.0 (40)	50 (50)	85	33	2	23	8	55	41
94	Atypical development	M	19	79 (10)	12.0 (50)	50 (80)	95	49	9	25	15	NA	42
102	Autism	M	28	101 (>99)	16.9 (97)	46 (<2)	65	67	11	34	22	55	53
113	Atypical development	F	26	91 (60)	13.4 (60)	49 (50)	75	8	0	8	0	65	40
120	Atypical development; autism	M	36	101 (93)	17.0 (93)	53 (98)	80	40	5	11	24	96	NI
144	Atypical development	M	18	80 (25)	9.0 (<2)	42 (<<2)	NA	0	0	0	0	NA	NI
146	Atypical development	M	34	84 (<2)	12.3 (10)	46 (<2)	NA	45	20	22	3	43	NI
165	Multiple congenital anomalies; atypical development	F	32	72 (<<2)	8.1 (<<2)	44 (<2)	55	20	9	9	2	15	NI
176	Moebius syndrome	M	37	97 (65)	13.7 (30)	41 (<<2)	55	71	13	39	19	19	NI
178	Atypical development	M	14	76 (50)	11.3 (50)	50 (98)	90	24	9	5	10	NA	NI
188	Atypical development	F	28	84 (11)	9.0 (<<2)	43 (<2)	NA	22	6	9	7	63	NI
218	Atypical development	M	23	88 (65)	14.0 (80)	51 (80)	95	43	7	13	23	80	NI
237	Atypical development	M	21	91 (97)	17.0 (>>99)	49 (75)	90	41	18	17	6	99	20

NA = not available/not performed due to patient noncompliance or time constraints, NI = not indicated, HT = height, WT = weight, and HC = head circumference.

BPI: the Behavior Problem Inventory (BPI-01) is a 49-item behavior-rating instrument with 14 specific self-injurious behavior, 24 stereotypic behavior, and 11 aggressive/destructive behavior items. Each of the three problem behavior groups is preceded by a generic definition that applies to all items within the group. Items are scored on a five-point frequency scale (0 = never, 1 = monthly, 2 = weekly, 3 = daily, and 4 = hourly) and a four-point degree-of-the-problem or severity scale (0 = no problem, 1 = a slight problem, 2 = a moderate problem, and 3 = a severe problem). Higher scores represent behavior problems for self-injurious behavior (SIB), stereotypy (Stereo.), or aggression (Aggr.).

BSID: Bayley Scales of Infant Development (mean ± SD = 100 ± 15).

CSBS: Communication and Symbolic Behavior Scales (mean ± SD = 100 ± 15).

CARS: Childhood Autism Rating Scale (>35 indicative of autism).

**Table 2:** Microarray results and interpretation of genetic findings.

Subject ID	Genetic category	Size	Number of genes	Candidate genes	Total number of ROHs/*F*	References
21	7q22.3 deletion	231 kb	2	*COG5 *	0 ROHs/—	IMGSAC, 2001 [18]
32	5p14.1 deletion	461 kb	1	*CDH9 *	0 ROHs/—	Wang et al., 2009 [19]
44	19p13.3 duplication 1p12 deletion	1043 kb 217 kb	48 1	*GAMT, NDUFS7 * *NOTCH2 *	3 ROHs/0.001 (unrelated)	Smeitink and van den Heuvel, 1999 [20] Caldeira-Ara$\overset{´}{\text{u}}$jo et al., 2005 [21] McDaniell et al., 2006 [22]
94	9q22.31 duplication 16p13.3 duplication Xp11.23 duplication	52 kb 86 kb 132 kb	1 5 7	*ROR2 * *CACNA1H * *WDR45, SYP *	2 ROHs/0.003 (unrelated)	Butler and Wadlington, 1987 [23] Splawski et al., 2006 [24] Saitsu et al., 2013 [25] Tarpey et al., 2009 [26]
102	22q11.21 duplication	131 kb	4	*PRODH *	4 ROHs/0.006 (unrelated)	Liu et al., 2002 [27]
113	Xq21.33 ROH Xq11.1 ROH Xp11.22-p11.21 ROH	4319 kb 514 kb 6237 kb	44 16 59	*PCDH19, SRPX2, OPHN1, SHROOM4, KDM5C, IQSEC2, SMC1A, HSD17B10, HWWE1, PHF8, FGD1 *	10 ROHs/0.013 (second-third cousins)	Roll et al., 2006 [28] Kasri et al., 2009 [29]
120	22q11.21 duplication	102 kb	2	*RTN4R *	10 ROHs/0.021∗ (first-second cousins)	Sinibaldi et al., 2004 [30]
144	None	—	—	*— *	2 ROHs/0.007 (unrelated)	
146	7q31.1 ROH	3436 kb	14	*FOXP2 *	4 ROHs/0.005 (unrelated)	Lai et al., 2001 [31]
165	20q13.2-q13.3 deletion	7338 kb	51	*GNAS, MC3R, CDH4, TFAP2C, ATP5E *	14 ROHs/0.029∗ (first-second cousins)	Butler, 2009 [32] Genevi$\overset{`}{\text{e}}$ve et al., 2005 [33]
176	3p12 ROH 17p11.2 ROH 22q13.1 ROH	3988 kb 4317 kb 4011 kb	8 64 68	*POU1F1 * *RAI1 * *EP300 *	7 ROHs/0.009 (second-third cousins)	Hendriks-Stegeman et al., 2001 [34] Girirajan et al., 2006 [35] Roelfsema et al., 2005 [36]
178	2q32.3 ROH 11q13.1-q13.3 ROH	3018 kb 3788 kb	19 94	*HSPD1 * *NDUFV1, NDUF58 *	8 ROHs/0.011 (second-third cousins)	Hansen et al., 2002 [37] Schuurs-Hoeijmakers et al., 2012 [38] B$\overset{´}{\text{e}}$nit et al., 2001 [39] Loeffen et al., 1998 [40]
188	1q21.1 duplication 22q11.21 duplication	1826 kb 102 kb	25 4	*NBPF9, PDE4DIP * *PRODH, RTN4R *	12 ROHs/0.021∗ (first-second cousins)	Sinibaldi et al., 2004 [30] Mefford et al., 2008 [41] Liu et al., 2002 [27]
218	3q26.31 duplication 9q21.33 ROH 17q22 ROH	74 kb 6070 kb 3220 kb	1 34 44	*NLGN1 * *NTRK2 * *MKS1 *	5 ROHs/0.007 (unrelated)	Auranen et al., 2002 [42] Glessner et al., 2009 [43]
237	7p22.3 duplication Xp21.1-p11.4 duplication	986 kb 7247 kb	15 26	*FAM2OC * *DMD, XK, OTC, * *TSPAN7, ATP6AP2, CHD19 *	0 ROHs/—	Manolakos et al., 2010 [44]

ROH = regions of homozygosity; *F* = coefficient of inbreeding (calculated for subjects with ROH >3 Mb in size); ^*^ *F* ≥ 0.0156 is categorized as consanguineous. Several genomic regions with losses and gains noted per subject were not included in this table. Only losses or gains were included for genes of known significance causing atypical development, behavior findings, or autism. All ROHs >3 Mb in size were included containing genes when disturbed are known to cause atypical development or behavioral problems. All deletions and duplications seen in our subjects represented heterozygous or one copy abnormalities.
